# Morphological Observation and Transcriptome Analysis of Ciliogenesis in *Urechis unicinctus* (Annelida, Echiura)

**DOI:** 10.3390/ijms241411537

**Published:** 2023-07-16

**Authors:** Dexu Kong, Maokai Wei, Danwen Liu, Zhengrui Zhang, Yubin Ma, Zhifeng Zhang

**Affiliations:** 1 Ministry of Education Key Laboratory of Marine Genetics and Breeding, College of Marine Life Sciences, Ocean University of China, Qingdao 266003, China; 18254308601@163.com (D.K.); maokaiwei1992@163.com (M.W.); lyhfanbin@sina.cn (D.L.); zhangzhengrui@ouc.edu.cn (Z.Z.); 2 Key Laboratory of Tropical Aquatic Germplasm of Hainan Province, Sanya Ocean Institute, Ocean University of China, Sanya 572000, China

**Keywords:** ciliary type, ciliogenesis, *Innexin*, *Urechis unicinctus*

## Abstract

During the early development of marine invertebrates, planktic larvae usually occur, and their body surfaces often form specific types of cilia that are involved in locomotion and feeding. The echiuran worm *Urechis unicinctus* sequentially undergoes the formation and disappearance of different types of body surface cilia during embryonic and larval development. The morphological characteristics and molecular mechanisms involved in the process remain unclear. In this study, we found that body surface cilia in *U. unicinctus* embryos and larvae can be distinguished into four types: body surface short cilia, apical tufts, circumoral cilia and telotrochs. Further, distribution and genesis of the body surface cilia were characterized using light microscope and electron microscope. To better understand the molecular mechanism during ciliogenesis, we revealed the embryonic and larval transcriptome profile of the key stages of ciliogenesis in *U. unicinctus* using RNA-Seq technology. A total of 29,158 differentially expressed genes (DEGs) were obtained from 24 cDNA libraries by RNA-Seq. KEGG pathway enrichment results showed that Notch, Wnt and Ca^2+^ signaling pathways were significantly enriched during the occurrence of apical tufts and circumoral cilia. Furthermore, all DEGs were classified according to their expression pattern, and DEGs with similar expression pattern were grouped into a module. All DEG co-expression modules were correlated with traits (body surface short cilia, apical tufts, circumoral cilia and telotrochs) by WGCNA, the results showed DEGs were divided into 13 modules by gene expression patterns and that the genes in No. 7, No. 8 and No. 10 modules were to be highly correlated with the occurrence of apical tufts, circumoral cilia and telotrochs. The top 10 hub genes in the above three modules were identified to be highly correlated with ciliogenesis, including the reported cilium-related gene *Cnbd2* and unreported cilium-related candidate genes *FAM181B*, *Capsl*, *Chst3*, *TMIE* and *Innexin*. Notably, *Innexin* was included in the top10 hub genes of the two modules (No. 7 and No. 8), suggesting that *Innexin* may play an important role in *U. unicinctus* apical tufts, circumoral cilia and telotrochs genesis. This study revealed the characteristics of ciliogenesis on the body surface of *U. unicinctus* embryos and larvae, providing basic data for exploring the molecular mechanism of ciliogenesis on the body surface.

## 1. Introduction

Cilia are membrane-like organelles that protrude from the surface of many eukaryotic cells and are widely distributed on the body surface of eukaryotic organisms and the internal and external surfaces of organs; they have the functions of locomotion, sensation, cell migration, signal transmission and nutrient transport (feeding) [1,2,3,4]. Currently, research on cilia in vertebrates is mostly focused on ciliogenesis and its related molecular regulatory mechanisms. Ciliogenesis is a multistep process [5], and migration of the centrosome to the cell surface represents the first regulatory event, during which the mother centriole forms a basal body, causing the ciliary axoneme to nucleate [6]. Axoneme extension represents the second regulatory event, during which distal structural vesicles (DAVs) accumulate and fuse to form larger ciliary vesicles (CVs). The third regulatory event is that microtubules between the basal body and CVs begin to assemble and extend into the axoneme, which pushes the CVs to move towards the cell surface and fuse with the cell membrane [7]. The ciliary membrane eventually wraps over the axoneme to form cilia [8]. In addition, ciliogenesis also involves multiple gene regulatory network processes. When the ciliogenesis signal is sent out, NEK kinase leaves the basal body to initiate ciliogenesis [9]. Subsequently, transcription factors, such as RFX and Foxj1, participate in the synthesis of related proteins during cilia assembly [10,11,12]. Finally, vesicular transport of ciliated membranes is initiated by IFT and Rab8a [13,14]. Ciliogenesis is a complex process of protein synthesis, transport, localization, and assembly, and is regulated by the Wnt signal pathway, Notch signal pathway, and Ca^2+^ signal pathway [15,16,17]. At present, research on the morphological structure, occurrence, and related molecular regulatory mechanisms of cilia has mainly focused on model organisms. Cilia also exist on the body surface of most marine invertebrates, exercising the functions of locomotion and feeding, but the molecular mechanism of ciliogenesis has rarely been studied.

During the early development of marine invertebrates, planktic larvae usually occur, and cilia of specific types are often formed on larval surfaces which play an important role in locomotion, sensing and feeding [18,19,20]. McDougall reported that cilia on the surface of polychaetes *Pomatoceros lamarckii* were first observed in blastulae [21]. In the early trochophore larvae, the apical tuft and prototroch appear, whereas in the middle trochophore larvae, the apical tuft disappears. At present, research on the body surface cilia of marine invertebrates has mainly focused on expression localization of cilium-related genes. In the annelid *P. lamarckii*, *α-tubulin*, *β-thymosin* and *caveolin* are mostly expressed in ciliated cells, such as ciliary band cells and apical tuft cells, suggesting that they may be involved in ciliogenesis [22]. In sea urchin *Strongylocentrotus purpuratus* embryos, *SoxB2* is neurogenic and involved in the regulation of oral and aboral ectoderm cilia [23]. Currently, there are few reports on the function of cilium-related genes on the body surface of marine invertebrates. *Calaxin* knockout was performed using CRISPR/Cas9 in the gastropod mollusk *Lottia goshimai*, and it was found that cilia on the body surface of trochophore larvae became shorter and locomotor ability was reduced [24]. In trochophore larvae of the bivalve *Mulinia lateralis*, the length and number of cilia were reduced by knockdown and knockout of *Cfap206* expression using RNAi and CRISPR/Cas9. Impaired sperm motility and abnormal sperm tail assembly were observed in male gonads, suggesting that *Cfap206* is essential for the formation and functionalization of cilia and sperm flagella in bivalve embryos [25]. So far, compared with model organisms, there are relatively few studies on ciliogenesis on body surfaces of marine invertebrates.

*Urechis unicinctus* belongs to the Echiuroidea (Xenopneusta, Urechidae), whose body surface is covered with cilia during development from the embryos to the trochophore larvae, and the body surface cilia gradually fall off during development from the segmentation larvae to worm-like larvae. The characteristics of cilia occurring and shedding on the *U. unicinctus* body surface provide ideal material for the study of ciliogenesis on the body surface. In this study, the type, structure and occurrence characteristics of body surface cilia in *U. unicinctus* embryos and larvae were identified. We then analyzed the transcriptomes of the key stages of body surface cilia formation and further identified the possible key genes for ciliogenesis on the body surface using weighted gene co-expression network analysis (WGCNA). This study systematically revealed for the first time the characteristics of ciliogenesis on the body surface of *U. unicinctus* embryos and larvae, and provides basic data for studying the molecular mechanism of the body surface cilia genesis of marine invertebrates.

## 2. Results

### 2.1. Observation of the Body Surface Cilia Distribution on the Embryos and Larvae of U. unicinctus

**Figure 1:**
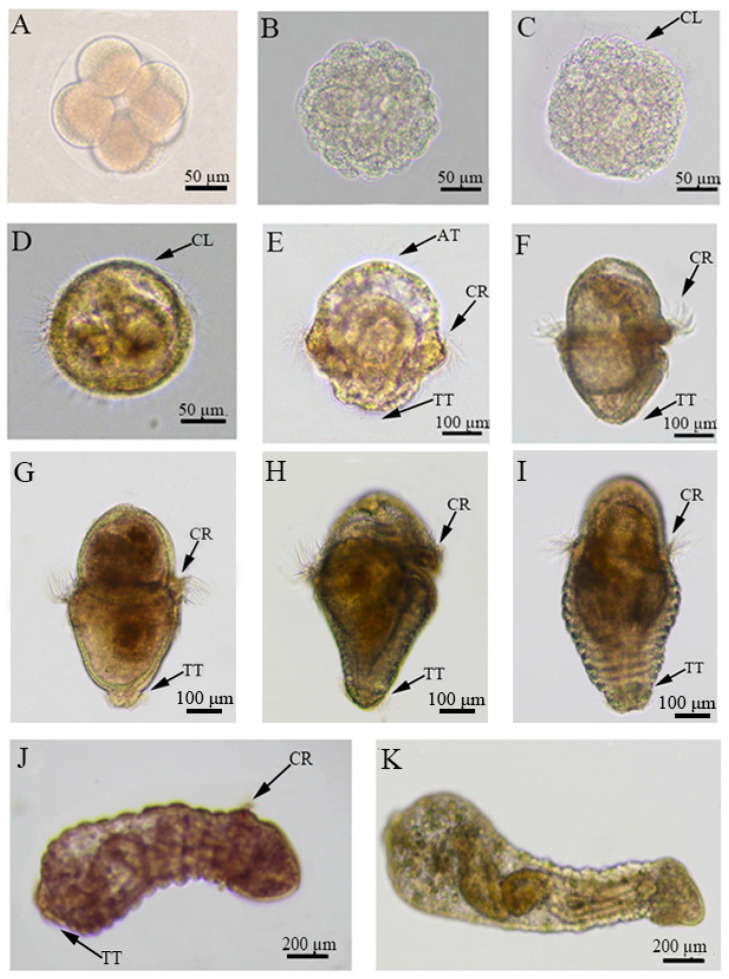
Morphological observation of the cilia distribution on the surface of embryos and larvae in *U. unicinctus*. (**A**) 8-cell embryo; (**B**) multicellular embryo; (**C**) blastula; (**D**) gastrula; (**E**) early trochophore larva; (**F**) middle trochophore larva; (**G**) late trochophore larva; (**H**) early segmentation larva; (**I**) middle segmentation larva; (**J**) late segmentation larva; (**K**) worm-like larva; CL: surface short cilia; AT: apical tuft; CR: circumoral ciliary ring; TT: telotroch.

Under light microscope, no visible body surface cilia were observed in embryos at each stage before blastula (14 hpf) of *U. unicinctus* (Figure 1A,B). When the embryos developed into blastulae, the body surface short cilia can be clearly seen (Figure 1C). The swing of these cilia allows the blastula to rotate slowly within the egg membrane. By gastrula (18 hpf), the density and length of the body surface short cilia further increased, but their distribution did not change (Figure 1D), and the embryo began to float upward. The distribution of short cilia on the body surface changed significantly after the gastrula hatched into trochophore larva. The early trochophore larva (24 hpf) has a variety of short cilia arranged on the body surface. The swing of these cilia can make the larva spiral in water. In addition to the ubiquitous short cilia on the surface, apical tufts of different lengths (7–16 μm) appear at the top of the upper hemisphere. In the equatorial region of the larva, cilia are densely arranged in a circle above the mouth cavity called the circumoral ciliary ring. The cilia at the bottom of the lower hemisphere of the larva were arranged to form a telotroch. The telotroch was shorter and less dense than circumoral cilia (Figure 1E). In the middle trochophore larva (48 hpf), the apical tuft degenerated, and other types of cilia remained unchanged (Figure 1F), at which time the larva began to feed. There was no significant change in body surface cilia during development from late trochophore larva (25 dpf) to middle segmentation larva (35 dpf) (Figure 1G–I). In the late segmentation larva (38 dpf), the body surface short cilia degenerated significantly, and the larva sometimes floated and sometimes crawled on the substrate surface (Figure 1J). After a brief crawling period on the substrate surface, the larva burrowed into the substrate and became worm-like larva (45 dpf). At this stage, all types of body surface cilia such as short cilia, circumoral cilia and telotroch degenerated (Figure 1K), and the larva lived in the bottom-cave.

### 2.2. Scanning Electron Microscopic Observation of Ciliogenesis in Embryos and Larvae of U. unicinctus

**Figure 2:**
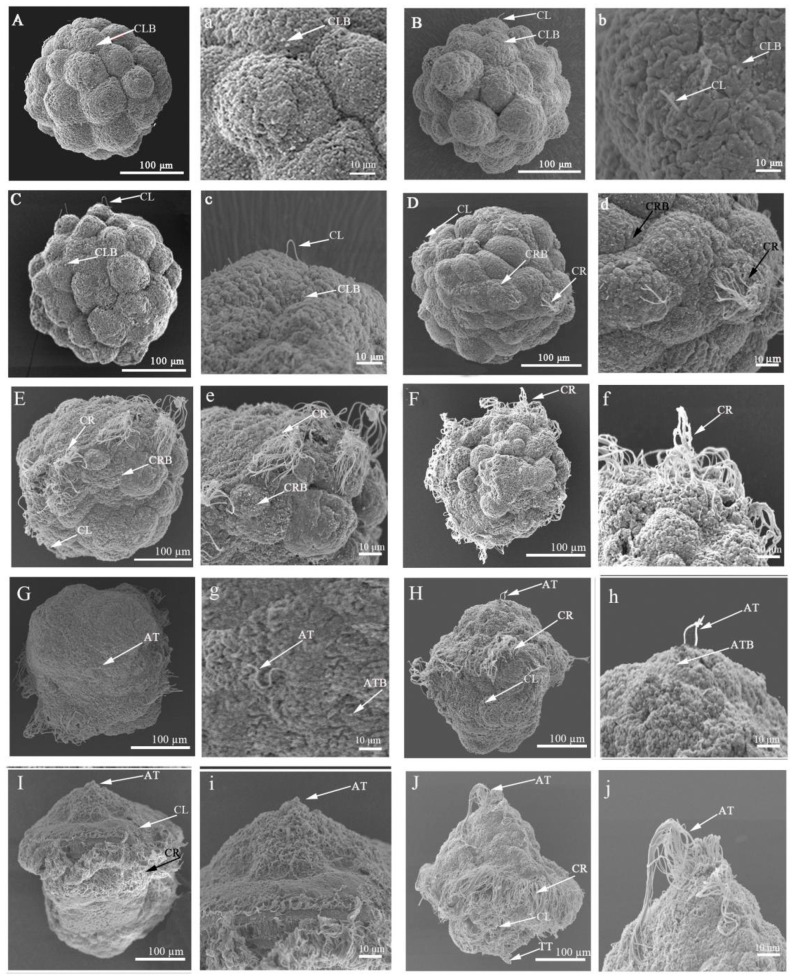
Genesis of the body surface cilia in *U. unicinctus* embryos and larvae observed by scanning electron microscope. (**A**,**B**) 32-cell embryos; (**C**,**D**) 64-cell embryos; (**E**,**F**) blastula; (**G**) gastrula; (**H**–**J**) early trochophore larva; (**a**–**j**) local magnification of the (**A**–**J**); CL: surface short cilia; CLB: surface short ciliated buds; CR: circumoral cilia ring; CRB: circumoral ciliated buds; AT: apical tuft; ATB: apical tuft buds; TT: telotroch.

Cilia on the body surface first appeared in the form of ciliary buds projecting from the cell surface, and they all originated from specialized multiciliated cells (Figure 2).

In 32-cell embryos (10 hpf), the ciliary buds were sporadically distributed on the surface of a few cells, with only 1–2 ciliary buds per cell surface (Figure 2A). After that, the number of ciliary buds increased and were scattered in the embryonic body, but the length of the mature ciliated body surface was shorter, usually between 12.5 and 22.6 μm (Figure 2B,C). These cilia are called body surface short cilia, and these body surface short cilia gradually increased and were irregularly distributed on the cell surfaces of the upper and lower hemispheres of the typical early trochophore larvae (Figure 2J).

In 64-cell embryos (11 hpf), ciliary buds began to appear on the cell surface in the middle of the embryonic body. These ciliary buds are precursors of circumoral cilia, whose length range is from 24.6 to 29.3 μm (Figure 2D). In the early blastula (14 hpf), the number of cells with circumoral ciliary buds increased in equatorial region of the embryonic body, and the number of ciliary buds on the surface of each cell reached 9–12 (Figure 2E). When entering a typical blastula (16 hpf), a circle of cells in the middle of the embryo was covered with circumoral cilia, and there were about 10–15 cilia per circumoral cilia cell, with the length between 31.5 and 40.6 μm (Figure 2F). In the typical early trochophore larva (24 hpf), the length of circumoral cilia increased significantly, reaching 53.6–64.4 μm, and formed a circumoral ciliary ring around the oral region of the larva (Figure 2J).

Until the gastrula (18 hpf), apical cilium buds appeared on the cell surface at the top of the embryonic body (Figure 2G). The number of apical cilia (buds) in the gastrula was very few, only 1–2 with a length of 3.5–5.5 μm, and then, the number increased to 2–3 per cell with a length of 5.5–8.5 μm in early trochophore larva (20 hpf) (Figure 2H). The density and length of the apical cilia increased with the development of the early trochophore larva (Figure 2I). In the typical early trochophore larva (24 hpf), the apical cilia with varying lengths clustered into an apical tuft with 15–20 cilia (Figure 2J).

Telotrochs were found at the end of the early trochophore larva, with a length of 11.5–20.2 μm (Figure 2J).

### 2.3. Transmission Electron Microscope Observation of Body Surface Cilia in U. unicinctus Larva

**Figure 3:**
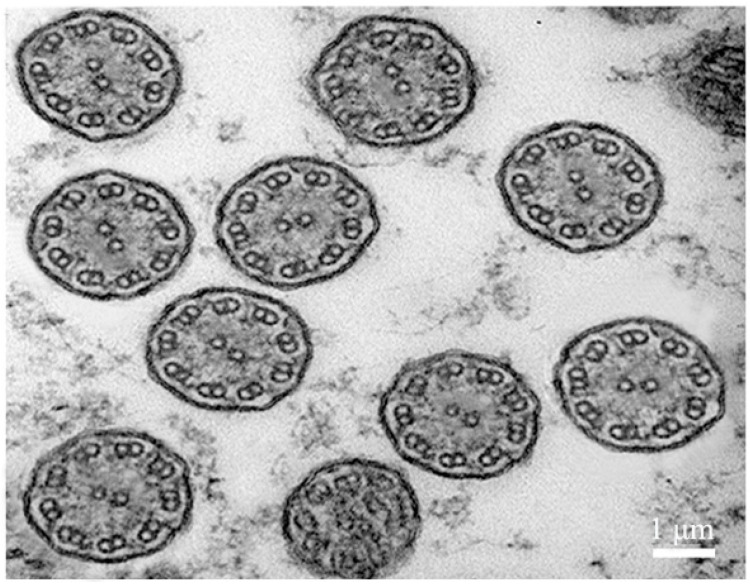
Transmission electron microscope observation of the body surface cilia in trochophore larva of *U. unicinctus*.

The body surface cilia in the early trochophore larva were complete, including the body surface short cilia, apical tuft, circumoral cilia and telotroch. Therefore, the early trochophore larvae (24 hpf) of *U. unicinctus* were selected as transmission electron microscope (TEM) samples to observe the structural characteristics of body surface cilia. The results showed that all types of the body surface cilia were “9 + 2” motile cilia (Figure 3).

### 2.4. Immunohistochemical Localization of Acetylated-α-Tubulin in Embryos and Larvae of U. unicinctus

**Figure 4:**
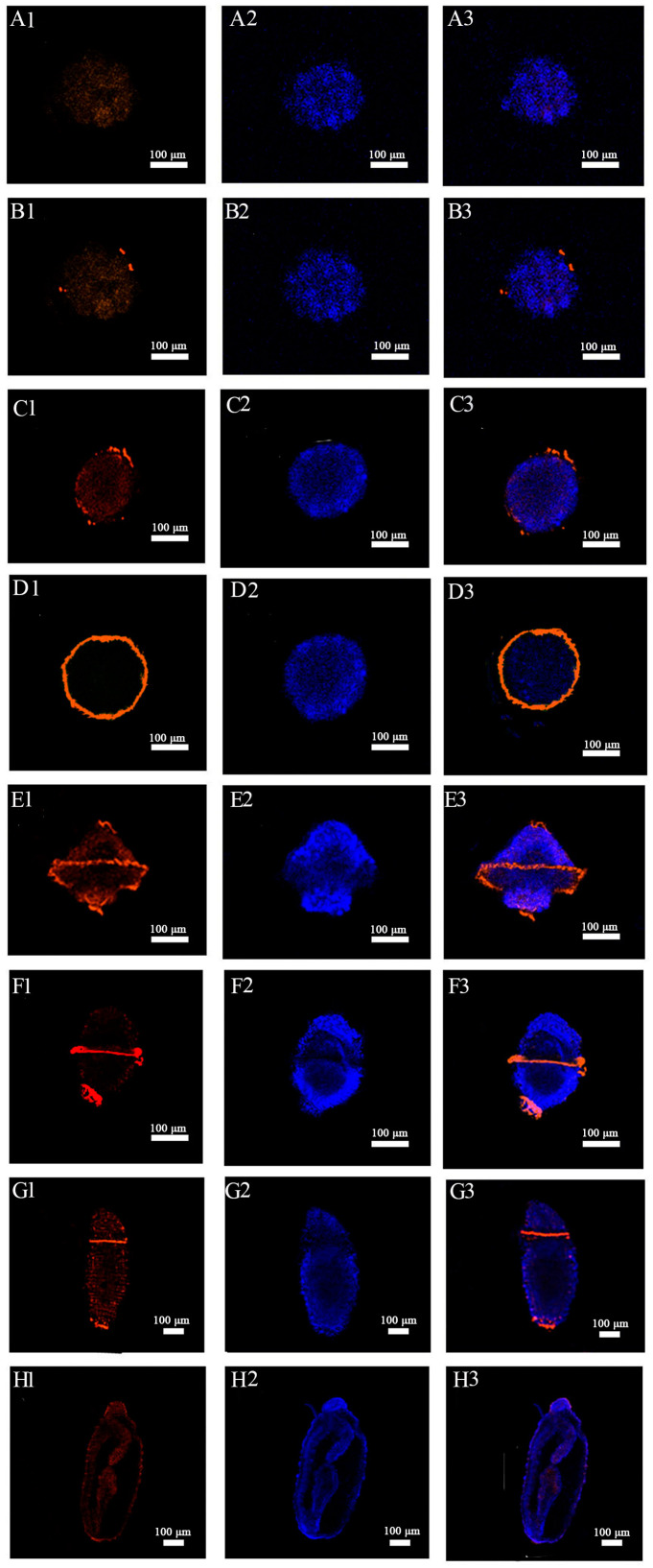
Cytological localization of ac-α-tubulin in embryos and larvae of *U. unicinctus*. (**A1**–**A3**) 8-cell embryos; (**B1**–**B3**) multicellular embryos; (**C1**–**C3**) blastula; (**D1**–**D3**) gastrula; (**E1**–**E3**) early trochophore larvae (side view); (**F1**–**F3**) middle trochophore larvae (side view); (**G1**–**G3**) late segmentation larvae (dorsal view); (**H1**–**H3**) worm-like larvae (side view); 1: red shows positive signal of ac-α-tubulin; 2: blue shows DAPI nuclear staining signal; 3: merge of 1 and 2.

To further characterize cilia, ciliated marker acetylated α-tubulin (ac-α-tubulin) was employed to verify the key stage of cilia formation in *U. unicinctus* embryos and larvae. The results showed that the positive signal of ac-α-tubulin was not detected before the multicellular embryos (10 hpf) (Figure 4A1–A3). The significant ac-α-tubulin positive signal first appeared in individual cells of multicellular embryo (12 hpf) (Figure 4B1–B3). In the blastula (14 hpf), the number of cells showing positive signal increased significantly (Figure 4C1–C3). In the gastrula (18 hpf), the positive signals were located in the circumoral ciliary ring cells around the embryonic body (Figure 4D1–D3). In early trochophore larva (24 hpf), ac-α-tubulin positive signals extended to apical tuft cells, circumoral ciliary ring cells and telotroch cells (Figure 4E1–E3). In the middle trochophore larva (48 hpf), the former apical tuft cells no longer showed positive signals, while the circumoral ciliary ring cells and telotroch cells were still detected with positive signals (Figure 4F1–F3). In the late segmentation larva (38 dpf), the number of circumoral ciliary cells and telotroch cells with positive signals reduced significantly (Figure 4G1–G3), and ac-α-tubulin positive signal was not visible in the worm-like larva (45 dpf) (Figure 4H1–H3). In conclusion, the pattern of ac-α-tubulin localization in *U. unicinctus* embryos and larvae is consistent with the morphological characteristics of the ciliogenesis on the body surface of embryos and larvae.

### 2.5. Illumina Sequencing, De Novo Assembly and Functional Annotation

To characterize the molecular mechanisms underlying ciliogenesis, RNA-Seq analysis of *U. unicinctus* embryos and larvae at key stages of ciliogenesis was performed in this study. A total of 24 cDNA libraries were constructed from embryos and larvae of eight developmental stages, 8-cell embryos (EC), multicellular embryos (MC), blastula (BL), gastrula (GA), early trochophore larva (ET), middle trochophore larva (MT), late segmentation larva (SL) and worm-like larva (WL). A total of 1,143,248,048 raw reads were generated, 1,142,760,492 clean reads were obtained and the total sequencing volume reached 171.4G (Table S1). The obtained clean reads were compared with the genome genes of *U. unicinctus*, more than 72% of clean reads in each library could be compared to corresponding genes in the genome, and the comparison rate to the unique location in the genome of *U. unicinctus* was greater than 64.6% (Table S2). Pearson correlation analysis showed that three biological replicates of each developmental stage sample were segregated together (Figure S1), indicating the reliability of the data.

### 2.6. Identification, Classification and Validation of the Differentially Expressed Genes (DEGs)

**Figure 5:**
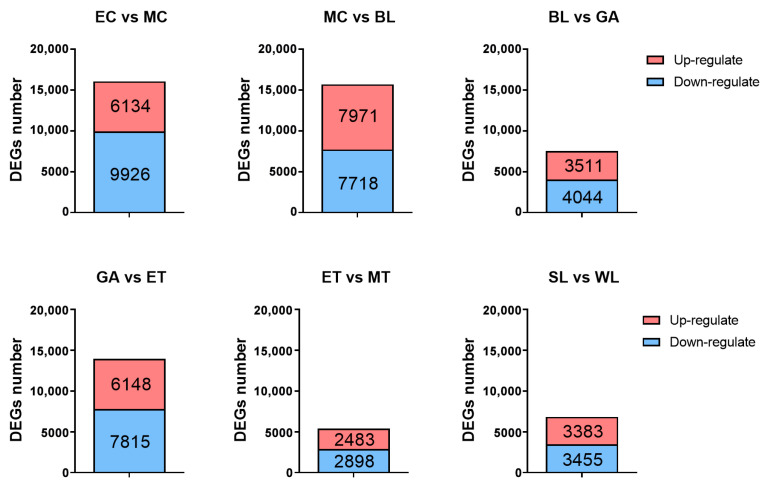
The number of differentially expressed genes (DEGs) between the transcriptomes of embryos and larvae at two adjacent developmental stages in *U. unicinctus*. EC: 8-cell embryos; MC: multicellular embryos; BL: blastulae; GA: gastrulae; ET: early trochophore larvae; MT: middle trochophore larvae; SL: late segmentation larvae; WL: worm-like larvae.

Pairwise comparisons of transcriptome data from samples of two adjacent development stages (EC vs. MC, MC vs. BL, BL vs. GA, GA vs. ET, ET vs. MT and SL vs. WL) were performed to screen the DEGs with the criteria of FDR ≤ 0.05 and |log2(fold change)| ≥ 1. A total of 29,158 DEGs were obtained (Figure 5), of which the number of DEGs among EC vs. MC, MC vs. BL, GA vs. ET was relatively high, at 16,060, 15,689 and 13,963, respectively. In the process from EC to MC, the body surface short cilia and circumoral cilia occur. In the process from MC to BL, the body surface short cilia and circumoral cilia grow intensively. In the process from GA to ET, telotrochs occur and circumoral cilia grow intensively, which is basically consistent with the early solid development process of the body surface cilia of *U. unicinctus* embryos and larvae. However, the number of DEGs between BL and GA, ET and MT as well as SL and WL were relatively low (7555, 5381 and 6838, respectively). It is preliminarily believed that this is not obvious with the morphological change in the body surface cilia of *U. unicinctus* at these stages, but the cilia of larvae are further developed and improved, which is consistent with the actual development process of embryos and larvae.

### 2.7. KEGG Enrichment Analysis Characterizing Possible Pathways Involved in Ciliogenesis

**Figure 6:**
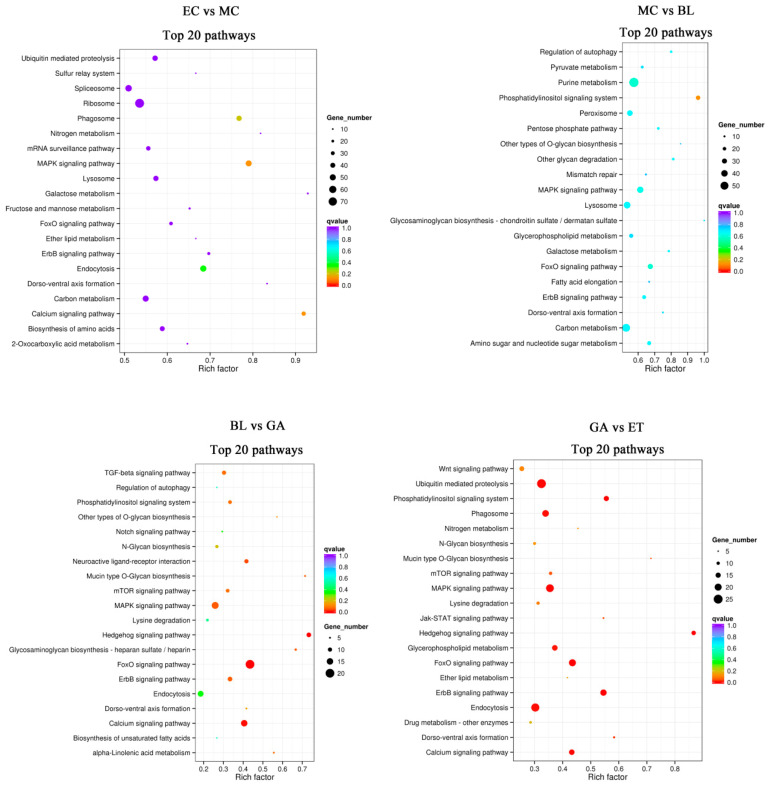

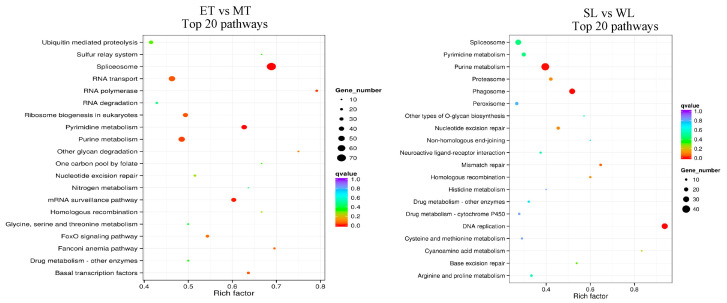
The top 20 pathways of KEGG pathway enrichment analysis from the differentially expressed genes between the transcriptomes of embryos and larvae at two adjacent developmental stages. EC: 8-cell embryos; MC: multicellular embryos; BL: blastulae; GA: gastrulae; ET: early trochophore larvae; MT: middle trochophore larvae; SL: late segmentation larvae; WL: worm-like larvae.

KEGG enrichment analysis was performed to determine the main signaling pathways of DEG enrichment during ciliogenesis in *U. unicinctus*. The results showed that the differentially expressed genes were significantly enriched in the development process, regulating growth-related signals, protein and RNA synthesis, modification, transport and degradation, and glycolipid metabolism (Figure 6). Among them, MAPK, ErbB and FoxO signaling pathways, amino acid and ribosome synthesis and Ca^2+^ signaling pathways related to ciliogenesis were the main enrichment pathway in the development of EC vs. MC. The main enrichment pathway of MC vs. BL was similar to that of EC vs. MC. Notably, in the DEGs of the BL vs. GA and GA vs. ET groups, some ciliogenesis-related pathways (Notch, Wnt and Ca^2+^) were significantly enriched. In addition, several protein synthesis and growth-related pathways (MAPK signal pathway, ErbB signal pathway, mTOR signal pathway, etc.) were also significantly enriched (Figure 6).

### 2.8. Expression Profiles of Genes Related to Ciliogenesis

**Figure 7:**
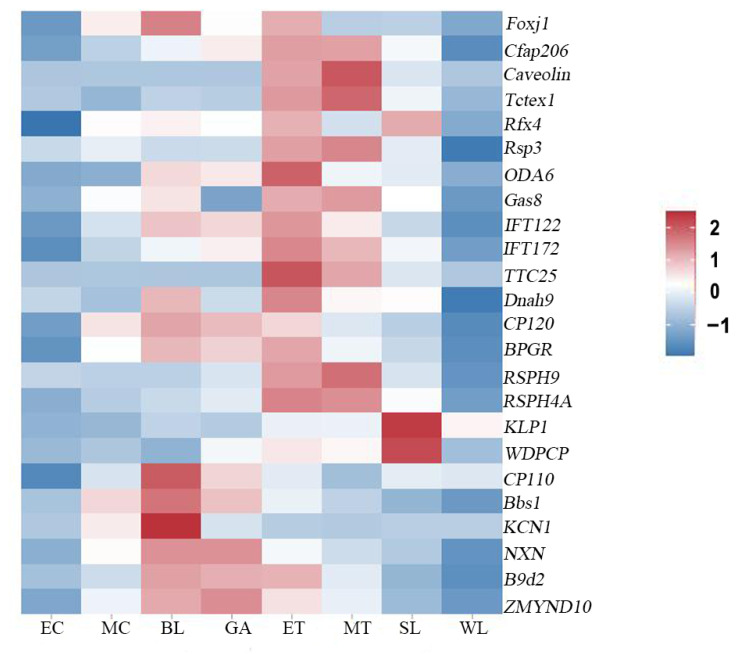
Hierarchical clustering of the cilium-related genes in *U. unicinctus* embryonic and larval transcriptomes. Foxj1: Forkhead box protein J1 [26]; Cfap206: Cilia and flagella-associated protein 206 [25]; Caveolin: Caveolin [27]; Tctex1: Dynein light-chain Tctex-type 1 [28]; RFX4: Regulatory factor X4 [29]; RSP3: Flagellar radial spoke protein 3 [30]; ODA6: 70-kD intermediate chain of flagellar outer arm dynein [31]; Gas8: Dynein regulatory complex subunit 8 [32]; IFT122: Intraflagellar transport protein 122 [33]; IFT127: Intraflagellar transport protein 127 [34]; TTC25: Tetratricopeptide repeat protein 25 [35]; Dnah9: Dynein axonemal heavy-chain 9 [36]; CP120: Centriolar coiled-coil protein of 120 [37]; RPGR: X-linked retinitis pigmentosa GTPase regulator [38,39]; RSPH9: Radial spoke head protein 9 homolog [40]; RSPH4A: Dynein regulatory complex subunit 4 [40]; KLP1: Kinesin-like protein 1 [41]; WDPCP: WD repeat-containing and planar cell polarity effector protein fritz homolog [42]; CP110: Centriolar coiled-coil protein of 110 [43]; Bbs1: Bardet–Biedl syndrome 1 protein [44]; KCN1: Small conductance calcium-activated potassium channel protein 1 [45]; NXN: Nucleoredoxin [46]; B9d2: B9 domain-containing protein 2 [47]; ZMYND10: Zinc finger MYND domain-containing protein 10 [48]; EC: 8-cell embryos; MC: multicellular embryos; BL: blastulae; GA: gastrulae; ET: early trochophore larvae; MT: middle trochophore larvae; SL: late segmentation larvae; WL: worm-like larvae.

To characterize the possible key regulatory genes during ciliogenesis, a total of 24 cilium-related genes were identified in the transcriptomes of *U. unicinctus* embryos and larvae (Table S3). These cilium-related genes were not detected in 8-cell embryos. In the beginning of multicellular embryos, several cilium-related genes (*Foxj1*, *CP120*, *Bbs1*, *KCN1*) were weakly expressed. In the blastulae, 14 ciliary genes were detected, of which 5 genes (*Foxj1*, *CP110*, *Bbs1*, *KCN1* and *NXN*) showed high expression. In the gastrulae, the expression abundance of cilium-related genes was generally weakened, and only that of *NXN*, *B9d2* and *ZMYND10* was maintained at the level of blastulae, suggesting that most cilium-related genes expressed in blastulae were ciliogenesis-related genes, while *NXN*, *B9d2* and *ZMYND10* might be related to the maintenance of ciliary function. In the early trochophore larvae, the expression abundance of these cilium-related genes generally increased, and most of the genes were expressed at a high level, of which expressions of *OD6A* and *TTC25* reached the peak, suggesting that most of the genes expressed in early trochophore larva were ciliogenesis-related genes. In the middle trochophore larvae, expression abundance of the cilium-related genes was higher, and the expression level of the gene *caveolin* reached its peak. In the late segmentation larva, the expression abundance of most ciliary genes decreased, except for those of *KLP1* and *WDPCP* were still at higher expression levels which might be related to the maintenance of ciliary function. In worm-like larvae, the expression abundance of all the ciliary genes decreased (Figure 7).

### 2.9. Weighted Gene Co-Expression Network Analysis Characterizing New Key Genes Involved in Ciliogenesis

**Figure 8:**
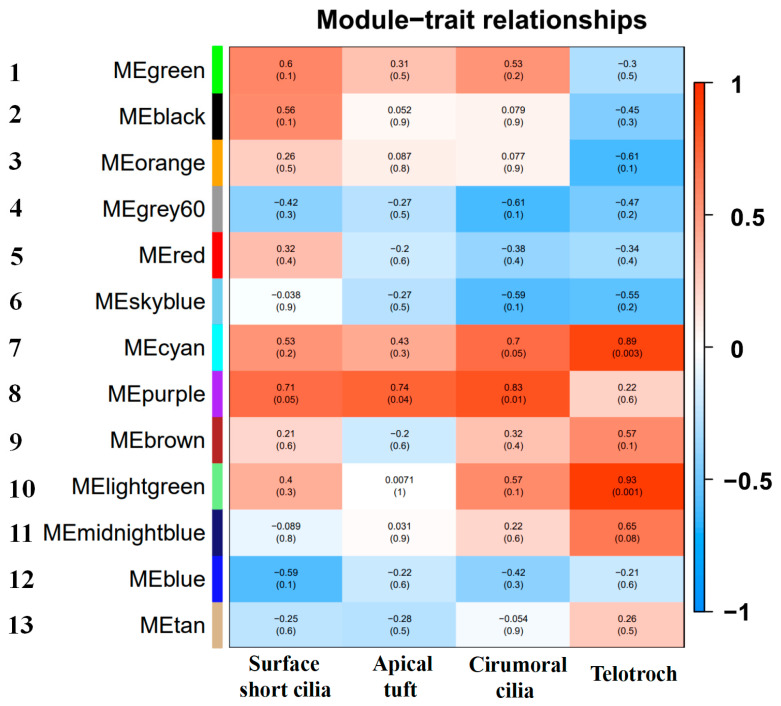
Clustering of correlations between gene co-expression modules and ciliogenesis traits. Each row with different color which is assigned arbitrarily by WGCNA on the left corresponds to a gene co-expression module, the numbers 1–13 represent 13 color modules from the top, where each column corresponds to a trait (body surface short cilia, apical tuft, circumoral cilia and telotroch); the background color in each case shows the correlation between the module genes and the traits, red represents a positive correlation, and blue is a negative correlation. The numbers in parentheses represent the significance (*p* value) between the modules and the traits.

To characterize the new key genes for the occurrence of body surface cilia in *U. unicinctus*, all DEGs were classified according to their expression pattern, and DEGs with similar expression pattern were grouped into a module. DEGs co-expression modules were correlated with traits (body surface short cilia, apical tuft, circumoral cilia and telotroch) by weighted gene co-expression network analysis (WGCNA). The results showed DEGs were divided into 13 modules by gene expression patterns (Figure 8). Among them, the No. 6 module had the lowest number of genes (129), while the No. 1 module had the largest number of genes (3253). Among the 13 co-expressed gene modules, the correlation between three modules (No. 7, No. 8 and No. 10) and traits was significant. Among these, genes in the No. 8 module were highly correlated with the apical tuft (*p* < 0.05) and circumoral cilia (*p* < 0.05), and the genes in the No. 7 module were correlated with circumoral cilia (*p* = 0.05), and the genes in the No. 7 and No. 10 modules were correlated with telotrochs (*p* < 0.05). Further, the hub genes in the three modules were characterized (Tables S4–S6). The top10 hub genes were mainly genes related to embryonic and organic development, cell metabolism and hormone regulation (Table 1, Table 2 and Table 3), and it was also found that the cilium-related gene *Cnbd2* was among them, and several top genes (*FAM181B*, *Capsl*, *Chst3*, *TMIE* and *Innexin*) that had not been reported related to ciliogenesis were identified. Notably, *Innexin*, a gap junction gene, was found in the top10 hub genes of the No. 7 module and No. 8 module.

## 3. Discussion

### 3.1. Similarities and Differences between the Body Surface Cilia of Embryos and Larvae from U. unicinctus and Other Lophotrochoza and Vertebrates

According to the spatial distribution of the body surface cilia in *U. unicinctus* embryos and larvae, it can be divided into body surface short cilia, apical cilia, circumoral cilia and telotrochs (Figure 1). Scanning electron microscopy (SEM) showed that the body surface short cilia of the embryos and larvae began from the ciliary bud, and the ciliary bud extended to form body surface short cilia. The body surface short ciliary buds first appeared on the surface of several cells in 32-cell embryo. During the blastula and gastrula, body surface short cilia gradually grew and were distributed in a dispersed pattern throughout the embryos. In early trochophore larva, body surface short cilia were distributed irregularly on the cell surface of the upper and lower hemisphere. Circumoral ciliary buds first occurred on the surface of the 64-cell embryo, and cells with distinct circumoral cilia formed a circle around the blastula. This ciliary distribution feature was also maintained in the gastrula, and the number and length of cilia in each circumoral ciliary cell increased compared to that of blastula. In the early trochophore larva, the cells with circumoral cilia located in the level of the mouth and encircled the middle of the larva. Apical ciliary buds first appeared sporadically on the surface of apical tuft cells in the gastrula, and apical tufts gradually formed in the early trochophore larva, as in other Lophotrochozoa [19]. The telotroch did not appear until the early trochophore larva. All body surface cilia in *U. unicinctus* embryos and larvae were generated in the form of ciliary buds that were projected from multiciliated cells (Figure 2). The body surface cilia in marine invertebrate larvae are of two types: pervasively distributed cilia and locally distributed ciliary rings, and the latter is usually denser than the former [49,50]. In *U. unicinctus* embryos and larvae, the ciliated system conforms this distribution rule.

Cilia on the body surface are common in the embryos and larvae of Lophotrochozoa, which acts as a moving organ to keep the larvae floating in water. In this study, body surface cilia were first observed in the multicellular embryo of *U. unicinctus*, and body surface cilia were clearly visible on the cell surface in the blastula. These cilia will form the body surface short cilia in the subsequent embryos and larvae, which also is a common feature in many marine invertebrates (annelids, bivalves and echinoderms, etc.) [21,51,52]. In Lophotrochozoa, the trochophore larva is a common, and usually contains various types of cilia on its body surface, such as apical cilia, prototrochs, metatrochs and telotrochs [53,54]. The apical tuft is located in the apical region of the early trochophore larva in polychaetes and bivalves, which senses the external environment and induces larval sedimentation [55]. In this study, SEM observations showed that the apical tuft of *U. unicinctus* appeared in the form of ciliary buds as early as the apical cells of the gastrula, and the apical tuft was formed in the early trochophore larva and then degenerated in the middle trochophore larva. In addition, the surface short cilia, apical tufts, and circumoral ciliary ring from *U. unicinctus* embryos and larvae occurred earlier than that of other Lophotrochozoa, which may be related to the research techniques. The genesis of various cilia in this study were obtained from SEM, whereas in other Lophotrochozoa, they were mostly based on light microscopy. Interestingly, the circumoral cilia in trochophore larva of lophotrochozoans is generally composed of two ciliary rings (prototroch and metatroch) around the mouth of the larva [56,57]. However, in *U. unicinctus* trochophore larva, only one circumoral ciliary ring was observed by light and SEM as well as immunohistochemical detection of ac-α-tubulin (Figure 1, Figure 2 and Figure 4). It needs to be further investigated whether this difference is caused by the different gene regulation pattern involved in the ciliogenesis.

The cilia on the body surface of *U. unicinctus* embryos and larvae function in locomotion and predation, which is consistent with that of other Lophotrochozoa. According to the observation results of transmission electron microscopy, the microtubule structure of the cilia on the body surface in the trochophore larva of *U. unicinctus* belonged to the “9 + 2” type, meaning these body surface cilia were the motile cilia (Figure 3), which are similar with the motile cilia in vertebrates and the cilia on the body surface lophotrochozoan larvae [49,50,58], indicating the evolutionary conservation of cilia in structure and functions whether the body surface cilia in Lophotrochozoa larvae or the mobile cilia in vertebrates.

### 3.2. Possible Key Genes and Pathways for Ciliogenesis on the Body Surface of Embryos and Larvae in U. unicinctus

A total of 24 cilium-related genes were identified in the embryo and larva transcriptome of *U. unicinctus* (Table S3), most of which were associated with cilia-assembly, wiggle, and generation. Gene hierarchical clustering analysis showed that the expression characteristics of most cilium-related genes in the embryos and larvae of *U. unicinctus* were consistent with the morphological changes in ciliary formation (Figure 7), suggesting that these cilium-related genes may be evolutionarily conserved in the formation and function maintenance of different types of cilia.

Wnt signaling pathway has been reported to be related to ciliogenesis [15]. In this study, the Wnt signaling pathway was ranking first in KEGG pathway enrichment between the transcriptomes of gastrula (GA) and early trochophore larva (ET) (Figure 6). During the development process from gastrula to early trochophore larva, apical tufts and telotrochs occurred and circumoral cilia were prolonged (Figure 2), and this suggests that the Wnt signaling pathway may be involved in regulating the occurrence of apical tufts and telotrochs as well as the elongation of circumoral cilia in *U. unicinctus* embryos and larvae. The Notch signaling pathway has also been reported to be related to ciliogenesis [16]. This signaling pathway was also significantly enriched during the developmental process from blastula (BL) to the gastrula (GA) (Figure 6). Due to the developmental process was associated with the occurrence of apical tuft and the elongation of circumoral cilia (Figure 2), it is speculated that the Notch signaling pathway may also participate in the regulation of apical cilia and elongation of circumoral cilia in *U. unicinctus*. In addition, this study also found that the Ca^2+^ signaling pathway reported to be related to ciliogenesis [17] was significantly enriched when embryos developed from 8-cell embryos to multicellular embryos (Figure 6), a key stage of surface short cilia and circumoral cilia development (Figure 2). Therefore, it is suggested that the Ca^2+^ signaling pathway may be involved in regulating the occurrence of surface short cilia and circumoral cilia in *U. unicinctus*. In addition, some pathways unreported to be related to ciliogenesis, including mTOR signaling, MAPK signaling and autophagy pathways, were enriched for the first time in the transcriptomes of *U. unicinctus* embryos and larvae at the key stages of ciliogenesis (Figure 6). It remains to be further verified by experiment whether these signaling pathways are involved in the ciliary formation.

WGCNA was used to analyze the correlation of DEGs traits (body surface short cilia, apical tuft, circumoral cilia and telotroch), and a total of 13 gene modules were obtained (Figure 8). By analyzing the hub genes that were highly correlated with ciliogenesis of the apical tuft, circumoral cilia and telotroch in two highly correlated No. 7 and No. 8 modules, we found that cilium-related genes *Cnbd2* ranked among them. Some genes, such as *FAM181B*, *Capsl*, *Chst3* and *Innexin,* were also ranked among the top 10; in particular, *Innexin* was included in the top10 hub genes of the two modules. Innexin, a transmembrane protein, is a unique protein that constitutes the gap junction semi-channel in invertebrates and plays a key role in embryonic development, organogenesis, cellular immune response and cell apoptosis [59,60,61,62,63]. Some studies have shown that *Innexin* also plays a key role in the regulation of microtubule assembly [64,65]. In our previous study, the *Innexin* mRNA was specifically localized in circumoral ciliary cells of *U. unicinctus* larvae [27]. These results suggest that *Innexin* may be a key gene related to ciliogenesis on the body surface of *U. unicinctus* embryos and larvae. In this study, the expression of the *Innexin* and Wnt signaling pathways was identified to be specified for ciliogenesis on the body surface in *U. unicinctus* embryos and larvae at the key stages. Invertebrate *Innexin* is the target gene of the Wnt signaling cascade, and the terminal molecule β-catenin in the Wnt signaling pathway initiates the transcription of *Innexin* by directly binding to the TCF site of Innexin [66,67]. These results suggest that *Innexin* may be a key gene for ciliogenesis on the body surface in *U. unicinctus* embryos and larvae.

## 4. Materials and Methods

### 4.1. Animals Collection and Larval Culture

Sexually mature *U. unicinctus* with 19 ± 0.4 cm in body length were collected from a coastal intertidal flat in Yantai, China. Mature sperms and oocytes were obtained by dissecting the nephridia (gonoducts) of the male and female worms, respectively. Artificial insemination was performed by mixing sperms and oocytes at a ratio of 10:1 in filtered seawater (FSW), and then the fertilized eggs were reared in FSW (19 °C, pH 8.3, and salinity 31‰). The embryos underwent cleavage and gastrulation within 24 h and then developed into early trochophore larvae. The larvae were fed with single-cell algae (*Chlorella vulgaris*, *Isochrysis galbana* and *Cheatoceros muelleri*). Embryos and larvae were collected. Parts of these samples were immediately frozen in liquid nitrogen and then stored at −80 °C for total RNA extraction. Other fixed samples of embryos and larvae at various developmental stages were sampled for subsequent experiments.

### 4.2. Sample Observation under an Optical Microscope

After fertilization, the embryos of *U. unicinctus* (fertilized eggs, 2-cell embryos, 4-cell embryos, 8-cell embryos, 16-cell embryos, multicellular embryos, blastocyst and gastrula) and larvae at various developmental stages (early trochophore larvae, middle trochophore larvae, late trochophore larvae, early segmentation larvae, middle segmentation larvae, late segmentation larvae and worm-like larvae) were collected, and the living larvae were moved into clean petri dishes, respectively. Nikon E80i microscope was used to observe whether the embryos and larvae at each developmental stage of *U. unicinctus* are moving, swimming, and the distribution characteristics of cilia. Embryos and larvae with typical cilia distribution characteristics were chosen to take pictures.

### 4.3. Scanning Electron Microscopy

Embryos (8-cell embryos, 16-cell embryos, multicellular embryos, blastocyst and gastrula) and early trochophore larvae of *U. unicinctus* were collected and fixed in 2.5% glutaraldehyde in 0.1 M phosphate-buffered saline (PBS) for 15 min at room temperature, then replaced with a new fixative for 30 min and then fixed with a new fixative at 4 °C overnight. Finally, the samples were fixed in a 1% osmic acid solution for 2 h. The samples were washed three times for 15 min in PBS and then dehydrated with serial methanol. The samples were treated with a mixture of ethanol and isoamyl acetate (1:1, *v*/*v*) for 30 min and then treated with isoamyl acetate for 2 h. The samples were dried using a carbon dioxide critical point dryer. After critical point drying, the samples were mounted on scanning electron microscopy (SEM) stubs, sputter-coated with gold and observed with a Philips XL30E scanning electron microscope to observe and take pictures on the machine.

### 4.4. Transmission Electron Microscope

2.5% glutaraldehyde and 1% osmic acid were used for double fixation of the early trochophore larvae (24 hpf), and the fixation method was the same as that used for the SEM samples. The samples were dehydrated using the SEM method described above, and then transferred from anhydrous ethanol to pure acetone for 20 min. The sample was immersed in a mixture of epoxy resin and acetone (1:1, *v*/*v*) for 1 h; then the mixture of epoxy resin and acetone (3:1, *v*/*v*) was treated for 3 h, overnight in epoxy resin at room temperature. The embedded sample was sliced by an ultra-thin microtome with a thickness of 80 nm. The sections were first stained with uranium acetate dye for 30 min, and then washed with double-steaming water. Then the slices were stained with lead citrate for 15 min and washed with double steaming water, the copper mesh with slices was placed in a clean petri dish for machine observation. Finally, a JEOL (Tokyo, Japan) JEM-1230 transmission electron microscope was used for observation and photography.

### 4.5. Immunohistochemistry (IHC)

Larvae were anesthetized with MgSO_4_·7H_2_O saturated solution for 10 min prior to fixation. Then, they were fixed in 4% paraformaldehyde for 16 h at 4 °C, and dehydrated with serial methanol (25%, 50%, 75% and 100%) in phosphate-buffered saline (PBS, pH 7.4) and finally stored in 100% methanol at −30 °C. Before immunohistochemistry, the embryo and larvae samples were rehydrated in a gradient methanol series (100%, 75%, 50% and 25%). Immunohistochemistry was conducted following the protocols described by Hou et al. 2020 [68], with the modification that sections were blocked with 3% bovine serum albumin (BSA) (Sangon Biotechnology, Shanghai, China) diluted with PBT (pH 7.4) for 1 h. Subsequently, the embryos and larvae were transferred to acetylated α-tubulin (ac-α-tubulin) antibody diluted 1:1000 in BSA and incubated overnight at 4 °C on a nutator. Embryos and larvae were then rinsed in PBT for 2 h and incubated with horseradish peroxidase (HRP)-conjugated goat anti-rabbit IgG antibody (Sangon Biotechnology, Shanghai, China) diluted 1:1000 in PBT for 2 h. Finally, the embryos and larvae were washed six times in PBT and incubated with 2.5% DAPI (Solarbio, Beijing, China) in the dark for 2 h to label cell nuclei. Observations and photographs were obtained using a Nikon E80i microscope (Nikon, Tokyo, Japan).

### 4.6. RNA Extraction and Illumina Sequencing

Total RNA was extracted from stored embryos (8-cell embryos (EC, 8 h post fertilization, hpf), multicellular embryos (MC, 12 hpf), blastocyst (BL, 14 hpf) and gastrula (GA, 18 hpf)) and larval (early trochophore larvae (ET, 24 hpf), middle trochophore larvae (MT, 2 days post fertilization, dpf), late segmentation larvae (SL, 38 dpf) and worm-like larvae (WL, 45 dpf)) sample using Trizol (Invitrogen, Carlsbad, CA, USA) following the manufacturer’s instructions. The concentration, purity, and integrity of the total RNA were analyzed using NanoDrop 2000 (Thermo Scientific, Wilmington, DE, USA) and 1.2% agarose gel electrophoresis. Three µg total RNA per sample was used for RNA-Seq library preparation. Sequencing libraries were generated using the NEBNext^®^ Ultra RNA Library Prep Kit for Illumina (NEB, San Diego, CA, USA) according to the manufacturer’s instructions. The mRNA with poly-A tail was isolated using Oligo-dT magnetic beads (Qiagen, Hilden, Germany) and then randomly broken into short fragments (250~300 bp) by adding fragmentation buffer. Random hexamer-primer was synthesized into first-strand cDNA using short fragments as templates. Subsequently, second-strand cDNA was synthesized using buffer, dNTPs, RNase H and DNA polymerase I. Short fragments were purified using the QiaQuick PCR extraction kit (Qiagen, Hilden, Germany) and washed with EB buffer for end reparation and poly(A) addition. The fragments were then ligated to sequencing adapters. After agarose gel electrophoresis, suitable fragments were selected as templates for PCR amplification to construct a cDNA library. Finally, these libraries (total 24 libraries from 24 RNA samples) were respectively sequenced with 150 bp paired-end reads using Illumina HiSeq X Ten system (Illumina, San Diego, CA, USA) by Novogene Company (Beijing, China).

### 4.7. De Novo Assembly and Function Annotation of Transcriptome

Raw reads were filtered to obtain high-quality reads (clean reads) based on the elimination of reads with connectors, poly-N and low-quality reads with a phred quality score < 20. Calculate Q20, Q30 and GC content of the filtered data. Filtered reads were aligned to the reference genome of *U. unicinctus* using HISAT 2.2.1 software (https://daehwankimlab.github.io/hisat2/, accessed on 5 October 2022), and the genes and transcripts of each sample were assembled and quantified using StringTi 2.2.0 software (http://ccb.jhu.edu/software/stringtie/, accessed on 20 October 2022). Gene expression levels were normalized to fragments per kilobase of transcript per million mapped reads (FPKM). A splicing database was generated based on the gene model annotation file. To determine the reliability of the results, Pearson correlation values between samples were calculated and sample correlation analyses were performed. The online software Evenn (http://www.ehbio.com/test/venn/#/, accessed on 13 November 2022) was used to draw a Wayne diagram.

### 4.8. Enrichment and Analysis of Differentially Expressed Genes (DEGs)

Differentially expressed genes (DEGs) between each of the embryonic and larval stages (EC vs. MC, MC vs. BL, BL vs. GA, GA vs. ET, ET vs. MT and SL vs. WL) were analyzed using the DESeq R software package, and DEGs were adjusted as FDR (False discovery rate) < 0.05, using Benjamini and Hochberg’s method. GO enrichment analysis of DEGs was performed using the ClusterProfiler R package, in which gene length deviations were corrected using the Wallenius non-central hypergeometric distribution model [69,70]. KEGG pathway enrichment analysis of DEGs was performed using the ClusterProfiler model [71]. Hierarchical cluster analysis was performed for the FPKM of 24 reported cilium-related genes identified from the transcriptome of *U. unicinctus* embryos and larvae to examine the similarity and diversity of these gene expression profiles. The heat map is displayed in the ‘heat map’ R-package.

### 4.9. Construction of Gene Co-Expression Network

Weighted gene co-expression network analysis (WGCNA) focuses on the correlation between gene expression and traits. Through hierarchical clustering, genes were divided into gene co-expression modules, with hub genes as the core, and the modules were further associated with traits to obtain relevant information. Co-expression network was constructed using the WGCNA package (https://cran.r-project.org/web/packages/WGCNA/index.html, accessed on 3 December 2022) in R [72]. In this study, the transcriptome data of *U. unicinctus* embryos and larvae including 8-cell embryos (8 hpf, no cilia), multicellular embryos (MC, 12 hpf, initial occurrence of body surface short cilia and circumoral cilia), blastula (BL, 14 hpf, intensive growth of body surface short cilia and circumoral cilia), gastrula (GA, 18 hpf, initial occurrence of apical tuft, body surface short cilia and circumoral cilia growing intensively), early trochophore larvae (ET, 24 hpf, presence of all four types of cilia), middle trochophore larvae (MT, 2 days post fertilization, dpf, disappearance of apical tuft, with continuous maintenance of body surface short cilia, circumoral cilia and telotroch), late segmentation larvae (LS, 38 dpf, disappearance of body surface short cilia, while continuous maintenance of circumoral cilia and telotroch) and worm-like larvae (WL, 45 dpf, disappearance of both circumoral cilia and telotroch) were selected as samples to construct a gene co-expression network with body surface short cilia, apical tuft, circumoral cilia and telotroch as four traits. A trait matrix was created to identify core genes highly associated with cilia using WGCNA. In this matrix, traits were numerically quantified; that is, samples without ciliated trait were labeled with the number “0”, while those with ciliated traits were labeled with the number “1” (Table S7). The correlation between characteristic genes and ciliary types was used to estimate module-trait association. The correlation between modules and traits was analyzed by “cor” function in R package statistics. The threshold value was set as 0.85, and if it was higher than the threshold, the genes were considered to be similar. Using the weighted value of the correlation coefficient, which means the gene correlation coefficient was raised to the NTH power. Based on the weighted correlation coefficient of this gene, the genes were classified according to the expression patterns, and the genes with similar expression patterns were grouped into a module. In addition, the “corPvalueStudent” function was used to calculate each relevant student’s asymptotic *p*-value via the R package WGCNA. A *p*-value < 0.05 indicated a significant correlation between modules and the traits. After genes were divided into different modules, module KME (eigengene-based connectivity) value was calculated using the signedKME function of WGCNA to measure the relationship between genes and modules. The larger the |KME| is, the more important the gene in the module network is.

## 5. Conclusions

In this study, we distinguished the types of body surface cilia (body surface short cilia, apical tufts, circumoral cilia and telotrochs) as well as their occurrence characteristics and distribution patterns in *U. unicinctus* embryos and larvae and found that all types of body surface cilia were produced from multiciliated cells. Furthermore, we presented the first transcriptome analysis focusing on *U. unicinctus* embryos and larvae at the key stages of ciliogenesis and identified 24 reported cilium-related genes, whose expression characteristics were consistent with the morphological changes of ciliogenesis in embryos and larvae of *U. unicinctus*. The Notch, Wnt and Ca^2+^ signaling pathways might involve in ciliogenesis in *U. unicinctus* embryos and larvae. *Innexin* may play an important role in ciliogenesis in the embryos and larvae of *U. unicinctus*. This study provides basic data on the molecular mechanisms of ciliogenesis in echiurans.

## Figures and Tables

**Table 1:** Top 10 hub genes in the No. 8 module.

Gene ID	Gene Name	KME	Gene Function
evm.model.Hic_asm_10.155	*FAM181B* (family with sequence similarity 181 member B)	0.995	Involved in embryonic development, meiosis, cell differentiation, nervous system and other processes.
evm.model.Hic_asm_6.1358	*TRP5* (Tetratricopeptide repeat protein 5)	0.993	Actin regulation, autophagy, and DNA repair.
evm.model.Hic_asm_14.687	*Innexin*	0.992	Development of embryos and organs.
evm.model.Hic_asm_2.868	*P450c17* (steroid 17 alpha-hydroxylase/17,20 lyase)	0.989	Involved in hormone synthesis.
evm.model.Hic_asm_10.436	E3 ubiquitin-protein ligase RBX1	0.989	Involved in embryonic development and cell survival.
evm.model.Hic_asm_8.163	*PCSK6* (Proprotein convertase subtilisin/kexin type 6)	0.988	Regulates cell proliferation and inflammatory response.
evm.model.Hic_asm_8.1080	*U2AF1L4* (Splicing factor *U2AF 26* kDa subunit)	0.988	Acts as a pre-mRNA splicing factor.
evm.model.Hic_asm_14.236.3	*UreD* (Urease accessory protein D)	0.987	Involved in activating urease apolipoprotein.
evm.model.Hic_asm_9.1210	*Capsl*	0.987	Development of embryos and organs.
evm.model.Hic_asm_10.721.1	*Cnbd2* (Cyclic nucleotide-binding domain-containing protein 2)	0.987	Controls the development of spermatoflagellar curvature

**Table 2:** Top 10 hub genes in the No. 7 module.

Gene ID	Gene Name	KME	Gene Function
evm.model.Hic_asm_12.772	*NACHRA10* (Neuronal acetylcholine receptor protein subunit alpha 10)	0.995	Involved in the regulation of auditory stimuli
evm.model.Hic_asm_13.362	*AP2S1* (AP-2 complex subunit sigma)	0.994	Involved in clathrin-dependent endocytosis.
evm.model.Hic_asm_13.51	*Mpv17-like protein*	0.993	Supports mitochondrial function.
evm.model.Hic_asm_0.559	*Innexin*	0.993	Development of embryos and organs.
evm.model.Hic_asm_3.848.1	*GSTs* (Glutathione S-transferase)	0.993	One of the most important metabolic enzymes in biotransformation.
evm.model.Hic_asm_10.1040	*SPATA18* (Mitochondria-eating protein)	0.991	Degrade damaged mitochondria and mitochondrial proteins.
evm.model.Hic_asm_4.311	*CHL1* (Neural cell adhesion molecule L1-like protein)	0.989	Regulates neuron migration, axon growth, and dendrite projection.
evm.model.Hic_asm_3.845	*SULT1B1*(Sulfotransferase Family 1B Member 1)	0.988	Ontogeny and hormone regulation.
evm.model.Hic_asm_1.737	*MDH1B* (Malate Dehydrogenase 1B)	0.988	Involved in the catalytic dehydrogenation of malate in the tricarboxylic acid cycle.
evm.model.Hic_asm_8.1588	*Chst3* (carbohydrate sulfotransferase 3)	0.988	Catalyzed proteoglycan sulfation.

**Table 3:** Top 10 hub genes in the No. 10 module.

Gene ID	Gene Name	KME	Gene Function
evm.model.Hic_asm_13.963	*LRRC74A* (Leucine-rich repeat-containing protein 74A)	0.997	The initiator of Met-1 or Met-27.
evm.model.Hic_asm_13.966	*TMIE* (Transmembrane inner ear expressed protein)	0.996	Play role in a cellular membrane location.
evm.model.Hic_asm_14.88	*FGL1* (Fibrinogen-like protein 1)	0.992	Immune-suppressive molecule.
evm.model.Hic_asm_0.423	*RAB8B* (Ras-related protein Rab-8B)	0.991	The small GTPases Rab are key regulators of intracellular membrane trafficking, from the formation of transport vesicles to their fusion with membranes.
evm.model.Hic_asm_13.1164	*Mcfd2* (Multiple coagulation factor deficiency protein 2 homolog)	0.990	The MCFD2-LMAN1 complex forms a specific cargo receptor for the ER-to-Golgi transport of selected proteins.
evm.model.Hic_asm_6.554	*Adcy8* (Adenylate cyclase type 2)	0.989	Converting ATP into cAMP triggers a cellular signaling response.
evm.model.Hic_asm_6.414	*VNN1* (Pantetheinase)	0.988	Amidohydrolase that hydrolyzes specifically one of the carboamide linkages in D-pantetheine, thus recycling pantothenic acid (vitamin B5) and releasing cysteamine.
evm.model.Hic_asm_14.1693	*Chrna3* (Neuronal acetylcholine receptor subunit alpha-3)	0.987	After binding acetylcholine, the AChR responds by an extensive change in conformation that affects all subunits and leads to opening of an ion-conducting channel across the plasma membrane.
evm.model.Hic_asm_15.505.1	*qvr* (Protein quiver)	0.987	Important regulator of the Sh K^+^ channel.
evm.model.Hic_asm_11.797	*DYDC2* (DPY30 domain-containing protein 2)	0.987	Functions as part of axonemal radial spoke complexes that play an important part in the motility of sperm and cilia (by similarity). Plays a crucial role during acrosome biogenesis.

## Data Availability

The RNA-Seq raw sequence data was submitted to the National Center for Biotechnology Information (NCBI) Sequence Read Archive database under the accession numbers SRR13480358, SRR13480357, SRR13480346, SRR13480335, SRR13480324, SRR13480313, SRR13480302, SRR13480291, SRR13480286, SRR13480285, SRR13480356, SRR13480355, SRR13480354, SRR13480353, SRR13480352, SRR13480351, SRR13480350, SRR13480349, SRR13480330, SRX4526075, SRX4526074, SRX4526081, SRX4526071 and SRX4526070.

## Supplementary Materials

The supporting information can be downloaded at: https://www.mdpi.com/article/10.3390/ijms241411537/s1.
