# Supplementary figures and images for: Morphological Observation and Transcriptome Analysis of Ciliogenesis in Urechis unicinctus (Annelida, Echiura)
Source: Int J Mol Sci. 2023 Jul 16;24(14):11537. doi: 10.3390/ijms241411537 (PMC10380512; doi:10.3390/ijms241411537)

EC

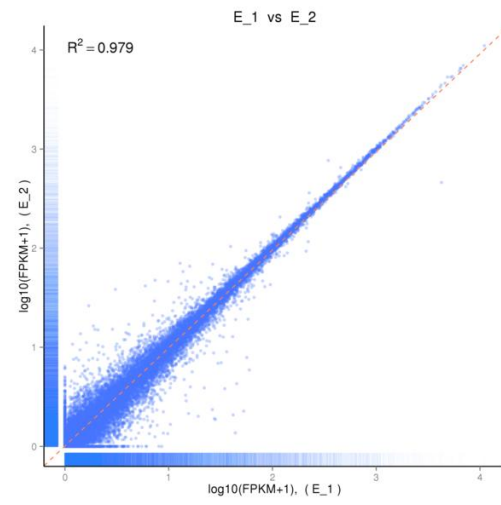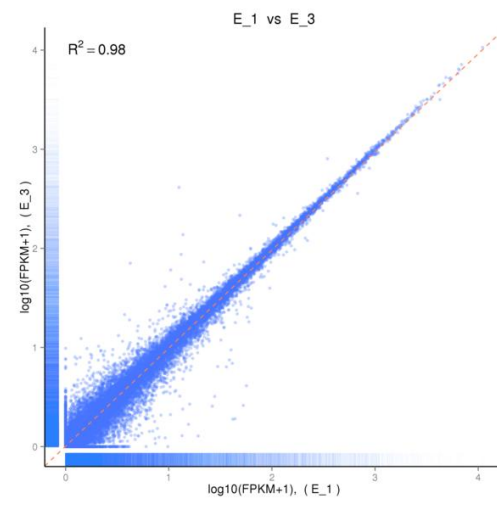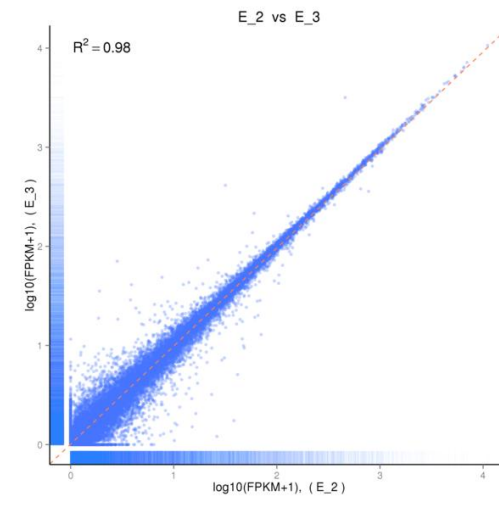

MC

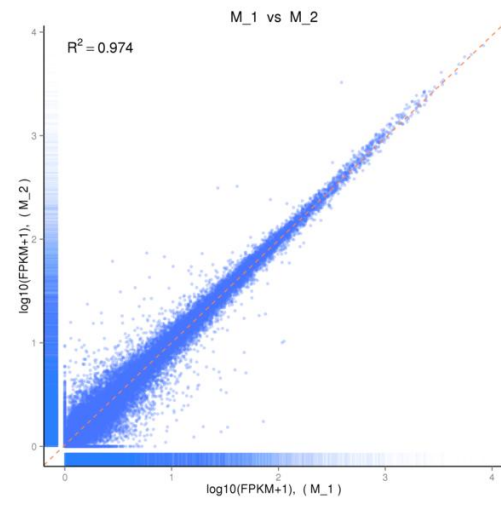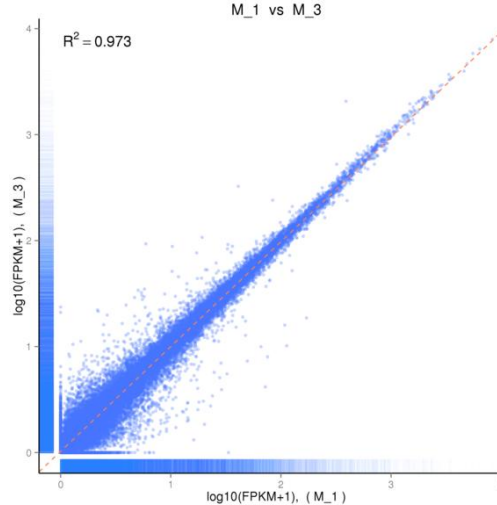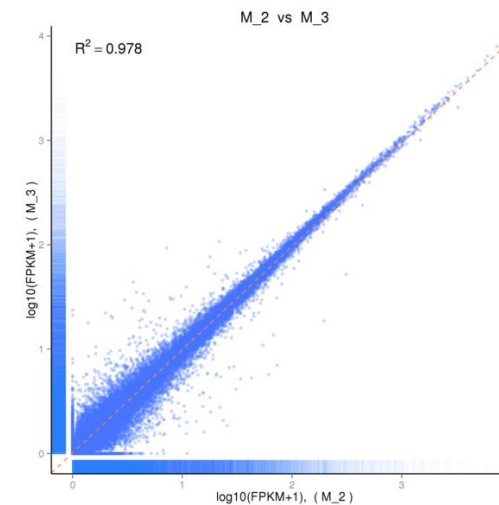

BL

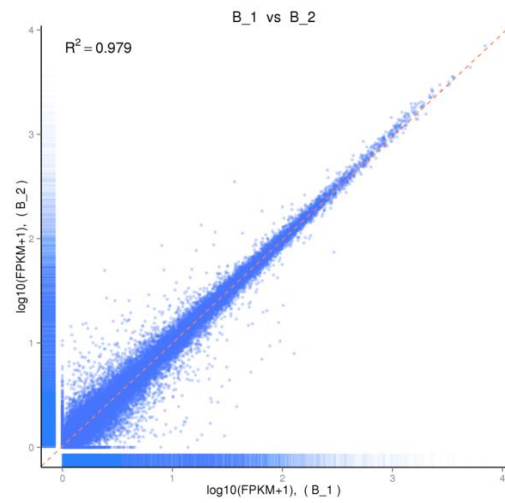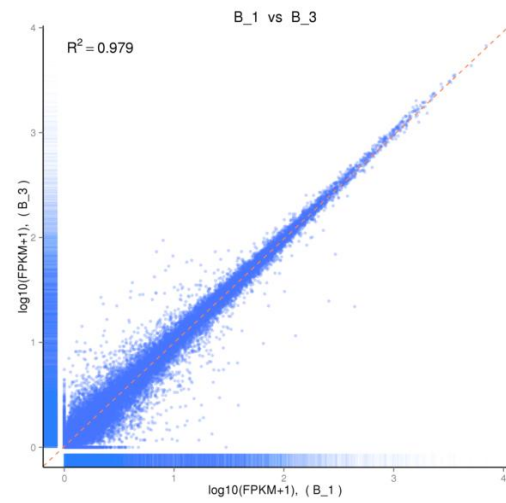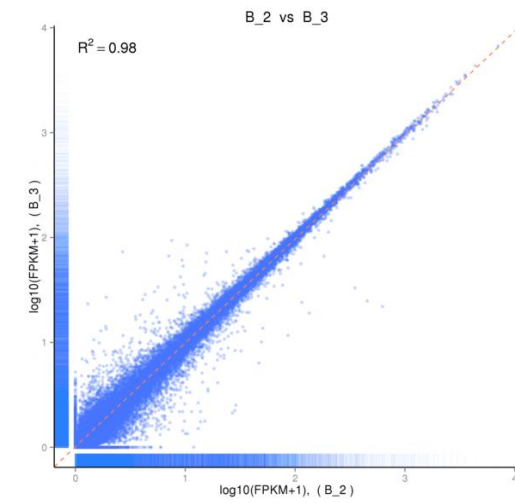

GA

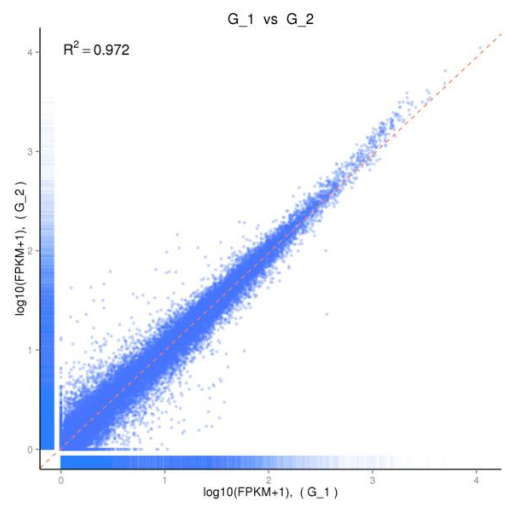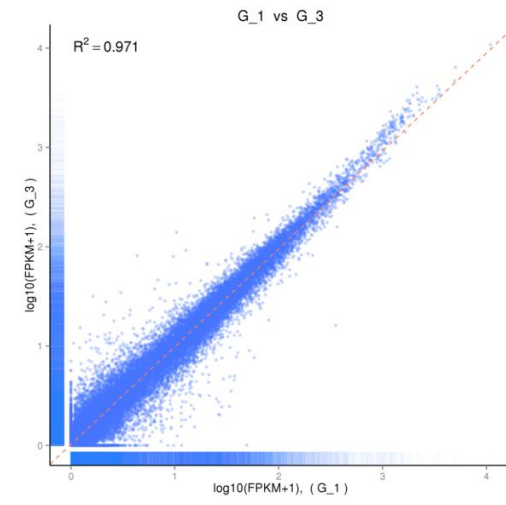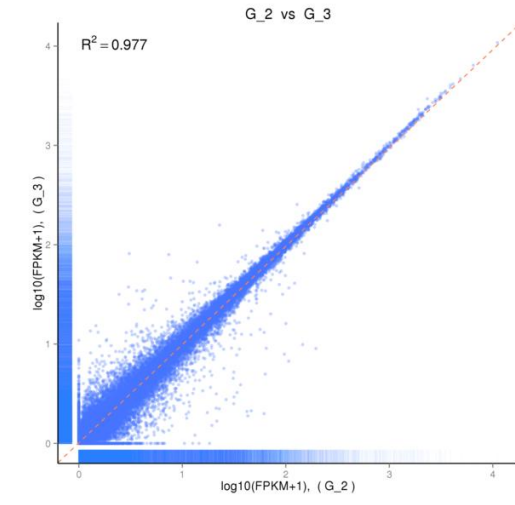

ET

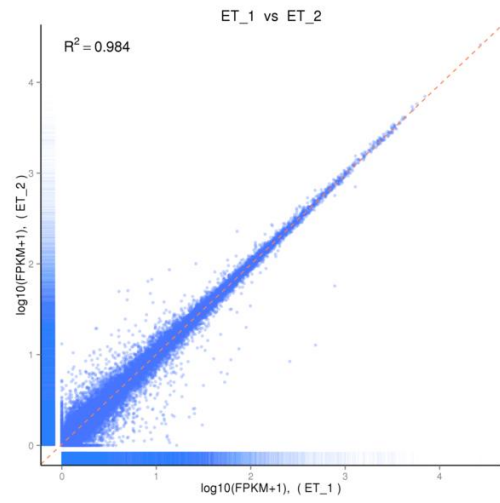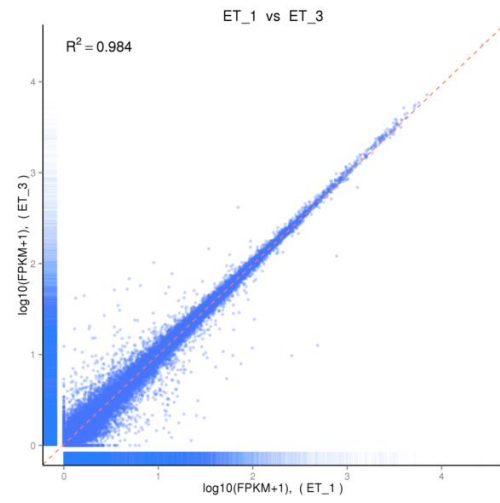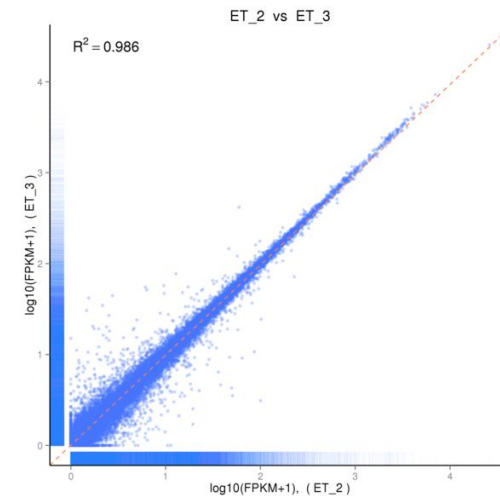

MT

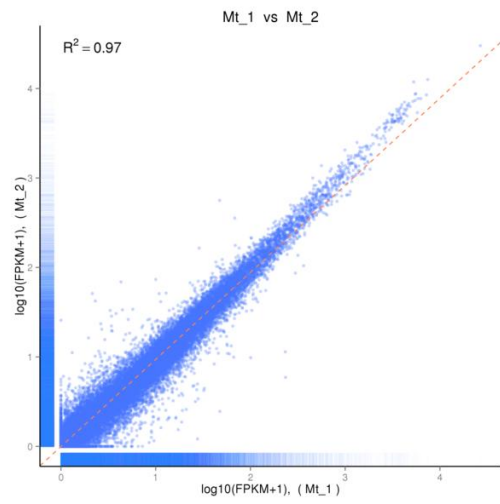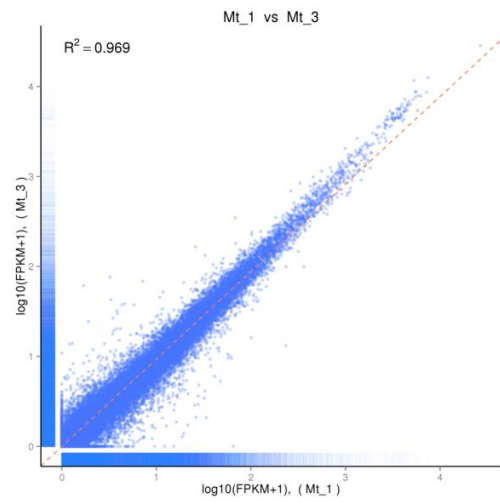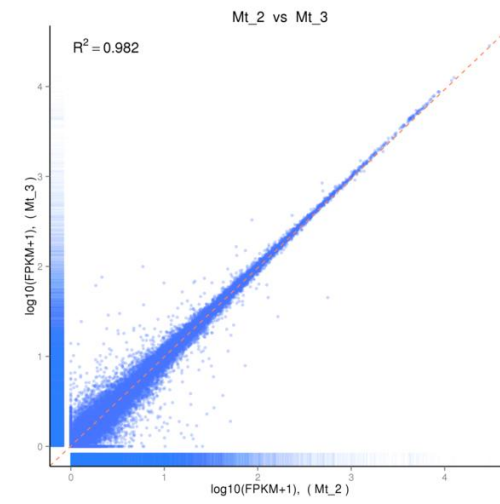

SL

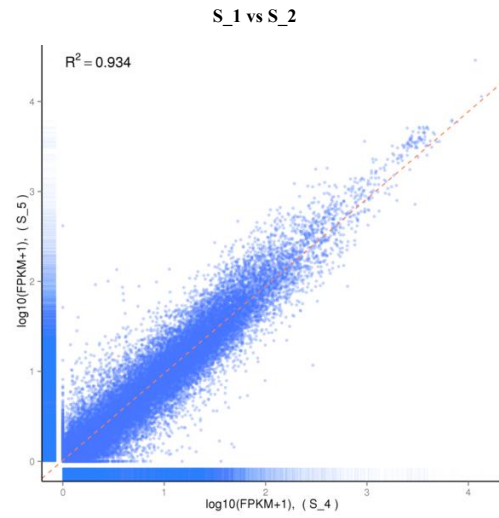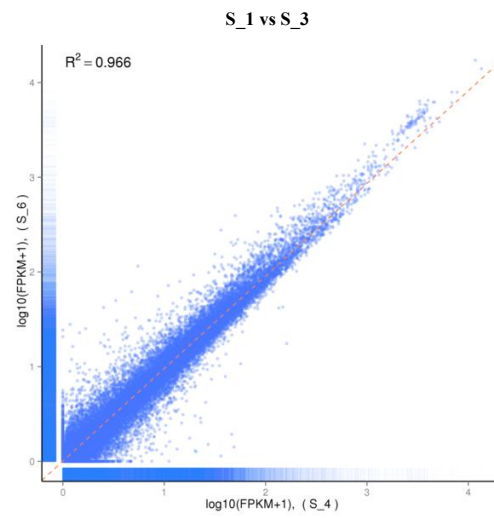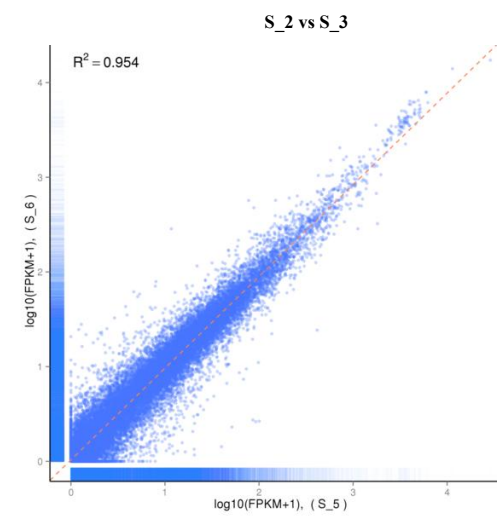

WL

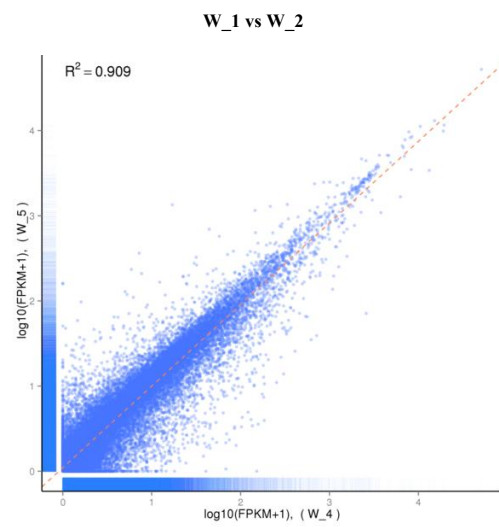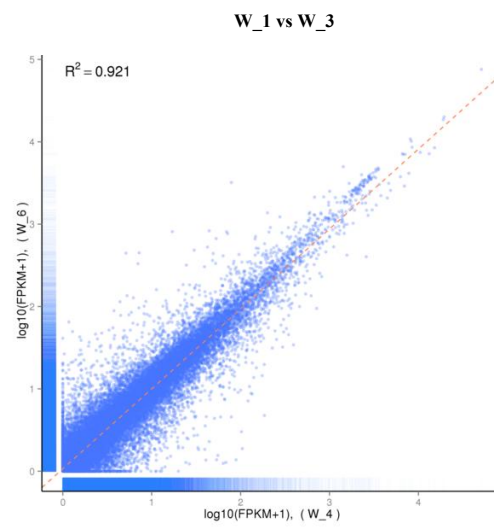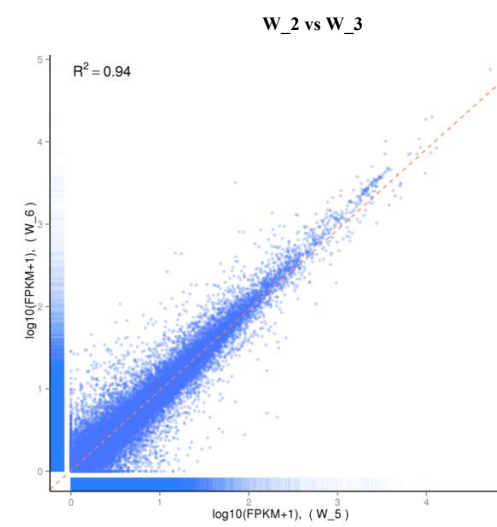

Supplement: Supplementary file 1 [file ijms-24-11537-s001.zip › Figure S1.pdf]
